# Plant Functional Groups Mediated the Effects of Plateau Pika Disturbance and Mowing on the Community Stability of Alpine Meadow Plants

**DOI:** 10.1002/ece3.73875

**Published:** 2026-06-30

**Authors:** Yu Chai, Chengyi Li, Xinru Du, Xilai Li

**Affiliations:** ^1^ College of Forestry and Grassland Qinghai University Xining Qinghai China; ^2^ State Key Laboratory of Plateau Ecology and Agriculture Qinghai University Xining Qinghai China; ^3^ College of Agriculture and Animal Husbandry Qinghai University Xining Qinghai China

**Keywords:** alpine meadows, community stability, external disturbances, plant functional groups, soil properties

## Abstract

The composition of plant functional groups is a primary factor influencing grassland ecosystem stability. Currently, it remains unclear how plant functional groups mediate the effects of external disturbances on the stability of alpine meadow ecosystems. This study aims to fill this knowledge gap via a six‐year controlled experiment involving three levels of mowing intensity (unmown, moderate, and heavy) and three levels of plateau pika ( *Ochotona curzoniae* ) density (0, 93.3, and 186.7 individuals·ha^−1^). Also explored is how they affected plant community composition, biomass by plant functional groups (grasses, sedges, and forbs) and their community stability, and soil properties, either individually or jointly. It is found that both types of disturbance significantly affected the importance value (IV) of plant functional groups. The medium‐density plateau pika and heavy mowing treatment reduced grass IV but increased forb IV. Both disturbances significantly affected plant functional groups' biomass and community stability, and their interaction significantly influenced the aboveground biomass (AGB) of grasses and sedges, as well as soil nutrients. Grass biomass decreased significantly following the disturbances, but forb biomass increased significantly. AGB of grasses and sedges exhibited a highly significant negative correlation with community stability. Conversely, AGB of forbs showed a positive correlation with community stability. Structural equation modeling (SEM) revealed that plant functional groups' biomass played a crucial mediating role in regulating the influence of plateau pika and mowing disturbances on plant community stability.

## Graphical abstract

The response mechanisms of plant community composition, functional group biomass, vegetation‐soil properties, and community stability to plateau pika and mowing disturbance intensity exhibited distinct differences. Gramineae and Sedge aboveground biomass and soil nutrient content demonstrated more pronounced responses to both disturbances and their interactions. Functional group biomass played a key mediating role in regulating the effects of plateau pika and mowing disturbances on plant community stability.

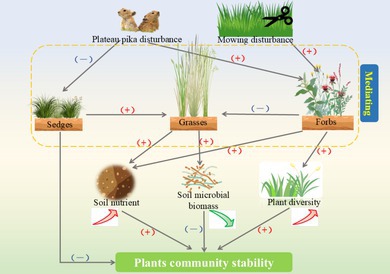

## 1 Introduction

Alpine meadows constitute a vital ecosystem on the Qinghai‐Tibet Plateau. Compared to forest ecosystems, they exhibit significantly lower stability, resistance to disturbances, and self‐repair capacity (Li, Yang, and Wang 2020). Over recent decades, driven by a warming‐drying climate and overgrazing, alpine meadow ecosystems have experienced varying degrees of degradation. Moderately to severely degraded grasslands now account for approximately 50% of the utilizable meadow area (Duan, Li, Li, et al. 2022). Degradation has significantly altered the structure and function of alpine meadow ecosystems, disrupting their inherent equilibrium. These changes manifest as declined grassland productivity, reduced species diversity, and shifted diversity and stability of plant functional groups (He et al. 2020). Ecosystem stability refers to the capacity of an ecosystem to maintain or restore its structure and function relative to a stable state following external disturbances (Jia et al. 2023) and is a fundamental attribute of every ecosystem. In this study, we specifically focused on community stability operationalized as temporal invariability, quantified by the inverse of the coefficient of variation (ICV) based on species population density. We chose density‐based invariability rather than biomass‐based stability because species density more sensitively reflects fine‐scale community structural shifts and interspecific replenishment under the dual‐factor interference of grazing and burrowing mammals in alpine meadows. The stability of the alpine meadow ecosystems is crucial for maintaining biodiversity on the Qinghai‐Tibet Plateau and predicting future ecosystem dynamics (Hillebrand et al. 2018).

As a valuable forage resource, the alpine meadow ecosystems on the Qinghai‐Tibet Plateau have been grazed for millennia by livestock. Grazing, as a traditional grassland management practice, has a variable ecological impact on meadow ecosystems, depending on its intensity and frequency. Moreover, in ecological research, mowing is frequently utilized as a standardized and controllable proxy for grazing to isolate the effects of biomass removal from other confounding factors such as trampling and nutrient return via excreta. Mowing, as a mechanical disturbance, exerts variable ecological impacts on meadow ecosystems depending on its intensity. Moderate mowing can enhance the photosynthetic efficiency of understory plants (Wang et al. 2025) and stimulate plant tillering, supporting the “compensatory growth” hypothesis in that community productivity temporarily increases following disturbance (Ma et al. 2010). Nevertheless, intensive mowing has destructive effects on ecosystems, especially over a prolonged period, leading to imbalances in plant community functional groups and reversing community succession (Ma et al. 2022). In meadow ecosystems, frequent or low‐stubble mowing can cause rapid degradation, reducing vegetation cover and height. The resultant short and sparse canopy is highly conducive to the invasion and thriving of plateau pikas, potentially leading to population outbreaks (Su et al. 2024). Conversely, if mowing is prohibited for a long time, the lack of external disturbance may lead to excessive competition among dominant species, resulting in declined biodiversity, slower nutrient cycling due to litter accumulation, and ultimately, a lower community productivity (Laia et al. 2022).

As the most dominant small herbivore and a keystone species on the Qinghai‐Tibet Plateau, the plateau pika ( *Ochotona curzoniae* ) plays a critical role in shaping ecosystem structure and function. Similar to mowing, plateau pika disturbance also exerts mixed effects on alpine meadows (Chai et al. 2024), depending on its intensity. Moderate plateau pika disturbance positively influences the ecosystem. Pika burrowing increases soil porosity, accelerates organic matter decomposition (Qin et al. 2021), and significantly enhances soil aeration and nutrient cycling, which, in turn, elicits increases in both aboveground and belowground vegetation biomass (Cheng et al. 2017). However, when pika population density exceeds the ecological threshold, the impact of their disturbance shifts towards negativity, such as a simplified plant community structure and a decline in functional redundancy, thereby threatening ecosystem stability (Qian et al. 2021). Jointly, the interactions of intensive mowing and pika disturbance change the competitive relationship between plant species and subsequently the composition and structure of the plant communities, exerting a negative impact on the soil and hydrology of alpine meadow simultaneously. Furthermore, both disturbances can jointly aggravate degradation and accelerate the degradation process of alpine meadow, and lower meadow ecosystem stability and the viability of certain functional plant groups (Liu et al. 2022).

In studying the impacts of disturbance on alpine meadow, current research predominantly treats either plateau pika or mowing disturbance in isolation, ignoring their nonlinear and potentially synergistic effects on alpine meadow community stability (Song et al. 2022). For instance, the reduction in vegetation cover following mowing facilitates concentrated foraging by pikas on the remaining biomass due to increased visibility and accessibility. Meanwhile, the physical root damage and soil loosening caused by pika burrowing may exacerbate the physiological stress on plants already weakened by aboveground biomass removal (simulated via mowing), thereby altering the overall community response (Su et al. 2024). How these cross‐scale coupling interactions affect meadow ecosystem stability and plant functional groups' viability remains unknown and urgently requires further quantitative research through controlled experiments.

Plant functional groups refer to a group of species within a community that share similar or identical functional traits. Alpine meadow plants can be classified into three functional groups according to their life form: grasses, sedges, and forbs based on clustered functional traits (Jiang et al. 2021). Dominant functional groups play a pivotal role in maintaining the temporal stability of community biomass (Ma et al. 2017). Furthermore, multiple functional groups suffer a significant loss in their biomass and a differential decline in various ecosystem functions if the ecosystem is severely disturbed (Li, Li, et al. 2021). However, current research focuses predominantly on dominant functional groups (such as grasses) only, while the contribution of other functional groups to ecosystem functions is either rarely studied or completely ignored (Mariotte et al. 2013). As far as stability maintenance mechanisms are concerned, the biomass redundancy hypothesis suggests that the coexistence of multiple functional groups can buffer the impacts of disturbance through functional complementarity (Hector and Bagchi 2007). Divergence among functional groups, facilitated by diversified resource acquisition strategies, can augment species diversity (Sanaei and Ali 2019), while also enhancing community resilience by increasing aboveground biomass (AGB) (Mariotte et al. 2013). Therefore, the coexistence of multifunctional groups can potentially buffer the effects of environmental variability on ecosystem functions, and plant functional groups are critical to determining overall community dynamics and resilience (Avolio et al. 2019). Consequently, an assessment of how plant functional groups influence the stability of alpine meadow ecosystems is of paramount importance for advancing our understanding of the drivers of grassland ecosystem functioning.

To fill the identified knowledge gaps, this study investigates and quantifies plant community composition, plant functional group biomass, and plant community stability in relation to soil properties. This study aims to address the following three questions: (1) How do vegetation community composition, functional group biomass, community stability, and soil properties change under the interactive disturbances of plateau pika and mowing? (2) How do vegetation and soil properties respond to different intensities of both sole and joint pika and mowing disturbances? And (3) In turn, how do vegetation and soil properties influence the stability of the degraded alpine meadow communities under the interactive disturbances of plateau pika and mowing? Based on these questions, we hypothesize that (1) Interactive disturbances from plateau pika and mowing will have a non‐linear effect on community stability; specifically, moderate intensities of both disturbances will enhance stability and diversity, while high‐intensity joint disturbances will significantly degrade soil properties and reduce stability; and (2) Plant functional group biomass mediates the influence of interactive plateau pika and mowing disturbances on alpine meadow community stability via several pathways, but chiefly by modulating plant diversity and soil properties. These hypotheses are tested in a moderately degraded alpine meadow in the source region of the Yellow River, Qinghai‐Tibet Plateau.

## 2 Materials and Methods

### 2.1 Study Area

The study area is situated in the Keqihetan floodplain (34°41′07″ N, 101°46′2″ E, average elevation: 3743 m), Henan Mongolian Autonomous County, in the eastern Three‐Rivers Source Zone of the Qinghai‐Tibet Plateau. Located 18 km from the county seat, this site is remote from human settlements. The predominant vegetation is of the alpine meadow type, and the region experiences a plateau continental climate, characterized by a mean annual precipitation of 615 mm and a mean growing season (May–October) temperature of 9.2°C–14.6°C. The study area, characterized by harsh climatic conditions and a complex eco‐environment, features extensive patches of moderately degraded alpine meadow with a distinct patchy degradation pattern. This degradation has been primarily driven by the synergistic effects of long‐term overgrazing, historical improper land management practices, and chronic disturbance by plateau pikas. In our study sites, mowing is the primary management intervention. Bare patches exceed 20% of the meadow area. Based on systematic taxonomy and widely recognized key functional differences among species, the plant community was categorized into three functional groups: grasses, sedges, and forbs (Hou et al. 2022). The dominant species in each group comprise *Carex alatauensis* and *Kobresia pygmaea* in sedges; *Elymus nutans* and *Poa pratensis* in grasses; and *Ajania tenuifolia*, *Ligularia virgaurea*, *Pleurospermum szechenyii*, *Saussurea pulchra*, among others, in forbs. A complete list of all the species and their respective functional groups is provided in Table S1.

### 2.2 Experimental Design and Sampling

**FIGURE 1:**
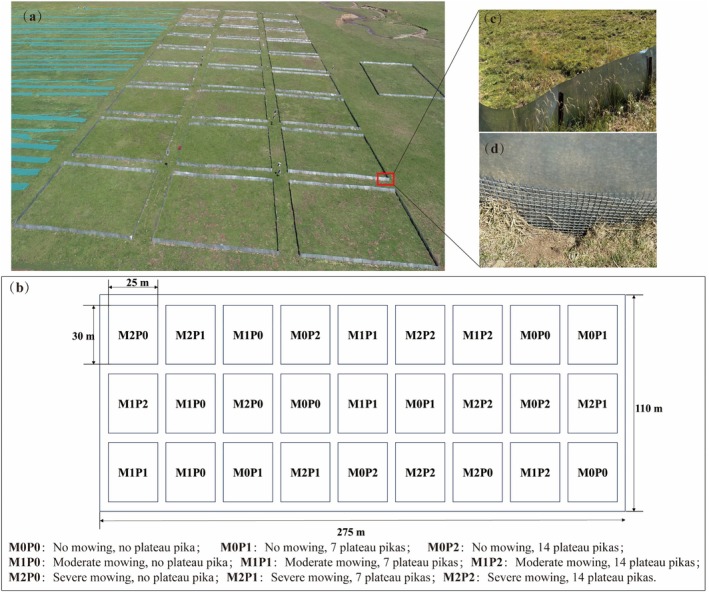
Aerial photograph providing a field overview of the experimental site in alpine meadow (a), schematic diagram illustrating the randomized block design and layout of the 27 experimental plots (b), the 0.6 m high above‐ground galvanized iron sheet fence installed to prevent pikas from roaming between adjacent plots (c), and the details of the customized steel wire mesh installed to a depth of 0.5 m below ground (mesh aperture: 2.5 × 2.5 cm) (d).

Experimental plots were established in August 2018, following a two‐factor, three‐level randomized block design, with plateau pika density and grazing (simulated via mowing) intensity serving as the two disturbances. Each was set at three levels (no for reference, moderate, and severe), leading to a total of nine treatments. Each treatment was replicated three times, resulting in 27 experimental plots (Figure 1a,b). These plots were arranged according to a factorial design incorporating three mowing intensities and plateau pika densities. To precisely control plateau pika density at different treatment levels, all experimental plots were enclosed with wire mesh fences. A plot dimension of 25 × 30 m was determined based on previous ecological studies indicating that this size exceeds the average home range of an individual plateau pika (Su et al. 2024), thereby ensuring that the animals could exhibit natural territorial and social behaviors without being constrained by the enclosed bounds. All adjacent plots were separated by a 5 m buffer corridor from each other. The experimental plots were fenced using customized steel wires. A rodent‐proof steel mesh (mesh aperture: 2.5 × 2.5 cm) was installed to a depth of 0.5 m below ground to prevent ingress and egress of plateau pikas and other herbivores (Su et al. 2024). Above ground, rodent‐proof galvanized iron sheets (0.6 m in height) were installed to prevent the enclosed pika from roaming between plots (Figure 1c,d). Angle irons (1.4 m length) at 2 m intervals were utilized to reinforce and stabilize the wire mesh fence (Song et al. 2025). Throughout the experimental period, the fence integrity was inspected monthly, and any damage was promptly repaired to ensure its effectiveness.

To ensure consistent pika densities within replicate plots, the number of pikas in each plot was strictly controlled in August every year from the established experimental plots by manually trapping all plateau pikas in all the plots. Subsequently, the captured pikas were released to each of them according to the prescribed research design at three quantities of 0 (reference), 7, and 14 individuals per plot, corresponding to a density of 0, 93.3, and 186.7 individuals·ha^−1^ (Li, Liang, et al. 2021), respectively. Specifically, no captured pikas were released to the plots designated as having a zero‐pika density (P0 as reference). Seven and 14 individuals were released to the medium‐density (P1) and high‐density (P2) plots, respectively, at a sex ratio of approximately 1:1. To maintain consistency in treatment intensity, pika population was annually censused across all the 27 plots each year since the experiment began, following the conclusion of their breeding season in August. Additionally, the “pika‐free” plots were inspected monthly and any intruding individuals were promptly removed to maintain the “no pika” state. All animal capture, handling, and release procedures in this study were reviewed and approved by the Science and Technology Ethics Committee of Qinghai University (Approval No. PJ202501‐161). The procedures were carried out in strict accordance with the approved protocol and relevant animal welfare regulations. During the annual census and relocation process, specialized live‐traps that minimize physical injury were used. Captured pikas were handled with care and held in well‐ventilated containers for the shortest duration possible (< 1 h) before being released back into their designated plots. No animal mortality or injury was recorded as a result of these handling procedures throughout the study period. Mowing intensity was established at three levels: no mowing (M0), moderate mowing (M1, grass clipped to a residual height of 12 cm, or approximately half the normal blade height), and heavy mowing (M2, grass cut to a residual height of 2 cm, the lowest height achievable by the mower blade). Mowing was carried out using a Honda HJ20‐Z pulse mower, and all mowed residue clips were removed from the plots. To simulate a typical forage utilization regime, a single mowing event was conducted annually during the peak of the plant growing season (August). This operation was performed consistently during the same period each year (2018–2023) to maintain treatment uniformity. The target stubble height was achieved by adjusting the mower blade height accordingly.

### 2.3 Measurement of Plant and Soil Properties

Vegetation and soil properties were surveyed or sampled within the 27 plots in August 2023 following six consecutive years of monitoring. Within each plot, three 0.5 × 0.5 m quadrats were randomly established as replicates, resulting in a total of 81 quadrats (27 plots × 3 quadrats). Although quadrats were randomly positioned, they were deliberately adjusted to avoid fresh pika mounds and active burrow entrances. This adjustment was necessary because these microsites often contain high concentrations of pika feces and unweathered subsoil. By avoiding these patches, we aimed to assess the representative state of the “background matrix” (the established vegetation and soil substrate) and ensure that the measured soil properties and plant community stability reflected the cumulative treatment effects across the plot, rather than transient, localized outliers. Within each quadrat, primary indicators of vegetation were surveyed, including species cover (visually estimated (Duan, Li, Chai, et al. 2022)), height (measured for 5–6 representative individuals per species), and density (determined by counting the number of individual stems or clumps for each species within the quadrat). Afterwards, all aboveground vegetation within the quadrat was clipped to the ground level, segregated by functional groups (grasses, sedges, forbs), and stored in labeled paper bags. In the laboratory, they were oven‐dried at 65°C to a constant weight. Dry weight was used to determine aboveground biomass (AGB) for each of the three functional groups and the whole community. Results originally obtained from the quadrats were subsequently aggregated to the plot level by averaging the results from the three replicate quadrats in each plot.

Following mowing, five soil samples (0–20 cm) were collected from each quadrat using a soil auger and homogenized to form a single composite sample. In total, 81 soil samples were collected. All composite samples were sieved (2 mm mesh) to remove roots, stones, and other debris, then transported to the laboratory for analyzing soil properties.

Soil organic carbon (SOC) content was determined using the potassium dichromate (K_2_Cr_2_O_7_)‐concentrated sulfuric acid (H_2_SO_4_) external heating method. Soil total nitrogen (STN) and total phosphorus (STP) were measured by concentrated H_2_SO_4_ digestion, followed by analysis using a Continuous Flow Injection Analyzer (AA3‐Auto Analyzer III, Germany). Soil microbial biomass carbon (MBC) and nitrogen (MBN) were extracted using the chloroform fumigation‐K_2_SO_4_ method, followed by determination using a TOC analyzer (Vario TOC, Germany). Soil microbial biomass phosphorus (MBP) was quantified using the molybdenum‐antimony anti‐spectrophotometric method.

### 2.4 Calculation of Plant Community Parameters

Several community parameters were calculated from the field‐surveyed data, including Importance Value (IV), diversity, evenness indices, and community stability. IV was selected as a comprehensive indicator reflecting the relative importance of species within the community and its degree of adaptation. It was calculated as follows:

(1) $$\mathrm{IV}=\left(\mathrm{RC}+\mathrm{RH}+\mathrm{RD}\right)/3$$

where RC refers to the cover (in %) of a particular plant species out of the total cover of all plant species; RH represents the height (in %) of a particular plant species out of the total height of all plant species; RD denotes the density (in %) of a particular plant species out of the total density of all plant species. Common species refer to species whose average importance value (IV) exceeds 10% in the same treatment. The 10% threshold was adopted to distinguish the primary functional species that consistently contribute to community structure and biomass from occasional or rare species. The IV of a functional group is the summed IV of all species of the same group.

The Shannon‐wiener index (*H*), Pielou's evenness index (E) in the α diversity measure were used to compare the changing characteristics of species diversity in each treatment. They were calculated as follows:

(2) $$H=-\sum \mathrm{Pi}{\mathrm{lnP}}_{i}$$

(3) E=H/lnS

(4) $$P_{i}=\frac{N_{i}}{N}$$

where *S* denotes the total number of species in a given plot; *N* is the total number of plant species in the same plot; *N*_*i*_ refers to the number of *i*th plant species; *P*_*i*_ is the proportion of the *i*th plant species to the total number of plant species.

Community stability was represented by inverting the coefficient of variation (ICV) of species population density, or

(5) ICV=μ/σ

where *μ* refers to the average density of each species in a given sample plot, and *σ* stands for the standard deviation of the density of each species. The higher the ICV value, the higher the community stability.

### 2.5 Statistical Analysis

Statistical analyses were performed using SPSS 26.0. Data normality was assessed using the K‐S test, and the homogeneity of variances was confirmed by Levene's test (*p* > 0.05). Given the 3 × 3 factorial randomized block design, two‐way ANOVA was employed to evaluate the main effects of mowing intensity (M), plateau pika density (P), and their interaction (M × *P*) on plant and soil characteristics. In this model, M and P were treated as fixed factors, while the “block” was included as a random factor to account for spatial heterogeneity. For significant effects, Duncan's multiple range test was employed for post hoc comparisons (*p* < 0.05). This method was chosen for its superior sensitivity in detecting treatment differences in complex factorial ecological experiments, following established practices in alpine meadow research. Correlation analyses and graphical visualizations were performed using Origin 2025. Redundancy Analysis (RDA) was performed using Canoco 5 software to identify key determinants influencing plant community stability. Structural equation modeling (SEM) was carried out using AMOS 29.0 to further analyze the direct and indirect pathways through which mowing and plateau pika disturbances influence plant community stability. An a priori model was established based on our theoretical framework, assuming that disturbances first alter the biomass of plant functional groups, which subsequently impacts community stability by modulating soil properties and plant diversity. Model fit was assessed using the following criteria: *χ* ^2^ test (*χ* ^2^/df < 3, *p* > 0.05), root mean square error of approximation (0 ≤ RMSEA ≤ 0.05), root mean square residual (0 ≤ RMR ≤ 0.05), comparative fit index (CFI ≥ 0.95), and goodness‐of‐fit index (GFI ≥ 0.95) (Yang et al. 2019). In the visualized results, only significant pathways (*p* < 0.05) were presented with standardized path coefficients to ensure visual clarity.

## 3 Results

### 3.1 Species Composition of Plant Community

**FIGURE 2:**
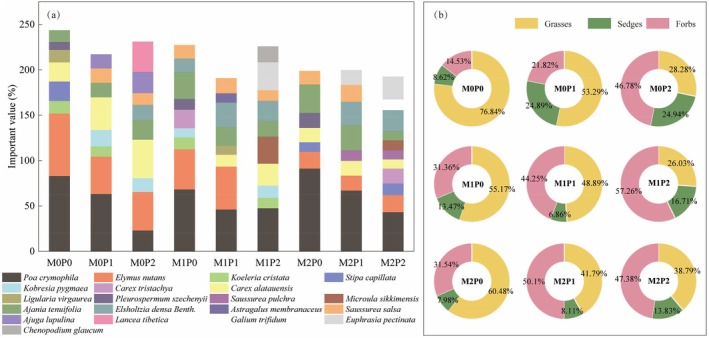
Important values of common species (a) and functional groups (b).

Field surveys of the vegetation community identified a total of 57 plant species (Table S1), of which 20 were classified as common species (Importance Value > 10%) (Figure 2a). Their distribution in the three functional groups was: 4 species constituting 20% of common species in grasses, 3 species constituting 15% of common species in sedges, and 13 species constituting 65% of common species in forbs. Dominant species exhibited consistent prominence across most treatments, such as *Poa crymophila* and *Elymus nutans* (grasses), along with *Carex alatauensis* in sedges. Their IV ranged from 22.88% to 91.09%, 16.64% to 68.90%, and 10.27% to 42.41%, respectively. In contrast, the dominant forb species varied considerably across different treatments.

At varying disturbance intensities, the proportional composition of the IVs of the plant functional groups shifted significantly (Figure 2b). At the same consistent mowing intensity, as plateau pika disturbance intensified, the proportion of grass plants gradually decreased, while the proportion of forbs gradually increased. The proportion of grasses' IVs in the plots severely disturbed by plateau pika (P2) decreased by 63.20% at no mowing, by 52.82% at moderate mowing, and by 35.86% at severe mowing in comparison to the reference (P0), and the proportion of forbs' IVs increased by 221.95%, 82.59%, and 50.22%, respectively, at the three mowing intensities.

### 3.2 Biomass of Plant Functional Groups and Community Stability

**FIGURE 3:**
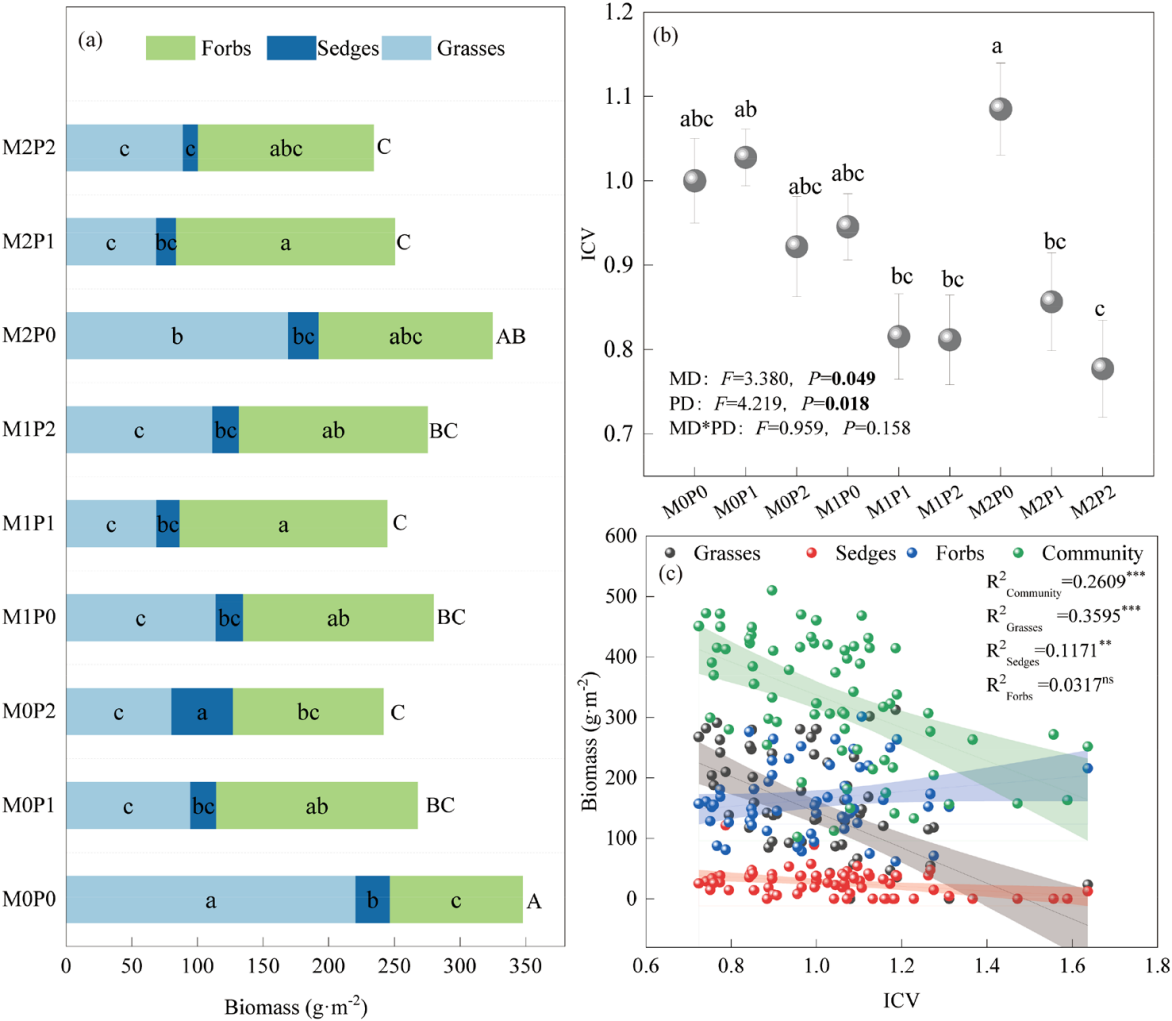
Biomass of plant functional groups (a), community stability (b) and correlation between aboveground biomass and stability (c) In Figure (a), different lowercase letters indicate that the biomass of the same functional group is significantly different between different treatments at the level of 0.05; Different lowercase letters indicate that the biomass of plant community is significantly different at the level of 0.05 among different treatments. 2. In Figure (b), different lowercase letters indicate that there are significant differences between different treatments of ICV at the level of 0.05.

Both mowing disturbance and plateau pika disturbance significantly impacted the biomass of all plant functional groups and their community stability (ICV). The interaction of the two disturbances had a significant effect on the aboveground biomass of grasses and sedges (*p* < 0.01) (Table 1). In the no‐disturbance treatment (M0P0), AGB_community_ and AGB_grasses_ reached 347.58 g·m^−2^ and 220.37 g·m^−2^, respectively, which were significantly higher than those in other treatments (*p* < 0.05), while AGB_forbs_ value was 100.96 g·m^−2^, which was significantly lower than in other treatments (*p* < 0.05). In the M2P2 treatment, both AGB_community_ and AGB_sedges_ reached 234.34 g·m^−2^ and 11.57 g·m^−2^, respectively, which were significantly lower than those in other treatments (*p* < 0.05, Figure 3a). Plant community stability (ICV) was significantly higher in the M2P0 treatment than in the other treatments (*p* < 0.05, Figure 3b). It reached the minimum value of 0.78 in the M2P2 treatment, representing a 27.78% decrease from that of the M2P0 treatment. Linear regression results indicated that ICV was significantly and negatively correlated with AGB_community_ and AGB_grasses_ (*p* < 0.001), significantly and negatively correlated with AGB_sedges_ (*p* < 0.01) (Figure 3c).

**TABLE 1:** Effects of mowing disturbance, plateau pika disturbance and their interactions on aboveground biomass, community stability and plant–soil properties.

	MD		PD		MD × PD	
	*F*	*p*	*F*	*p*	*F*	*p*
AGB_grasses_	3.573	**0.033**	24.955	**0.000**	4.744	**0.002**
AGB_sedges_	9.438	**0.000**	4.158	**0.019**	5.917	**0.000**
AGB_forbs_	3.640	**0.031**	6.886	**0.002**	0.675	0.611
AGB_community_	0.657	0.521	8.121	**0.001**	1.695	0.159
ICV	3.380	**0.046**	4.219	**0.018**	0.959	0.435
H	0.140	0.869	3.683	**0.041**	2.035	0.097
E	0.291	0.748	4.035	**0.021**	0.524	0.718
S	0.066	0.936	0.611	0.545	0.368	0.831
SOC	1.012	0.368	1.913	0.154	5.282	**0.001**
STN	1.662	0.196	2.082	0.131	13.389	**0.000**
STP	3.028	0.054	3.171	**0.047**	3.474	**0.011**
MBC	0.210	0.811	0.620	0.541	0.432	0.785
MBN	0.184	0.832	3.555	**0.045**	0.672	0.613
MBP	3.885	**0.040**	1.718	0.186	0.860	0.491

*Note:* Data indicate *F* value and *p* value, Boldfaced numbers indicate that the *p* value is less than 0.05.

Abbreviations: AGB_community_, Aboveground biomass of community; AGB_forbs_, Aboveground biomass of forbs; AGB_grasses_, Aboveground biomass of grasses; AGB_sedges_, Aboveground biomass of sedges; E, Pielou evenness index of plants; H, Shannon‐Wiener index of plants; ICV, Plant community stability; MBC, Soil microbial biomass carbon; MBN, Soil microbial biomass nitrogen; MBP, Soil microbial biomass phosphorus, the below is same; MD, mowing disturbance; PD, plateau pika disturbance, the below is same; S, Number of plant species; SOC, Soil organic carbon; STN, Soil total nitrogen; STP, Soil total phosphorus.

### 3.3 Plant and Soil Properties

Mowing disturbance had a significant effect on MBP (*p* < 0.05), while plateau pika disturbance had a significant impact on H, E, STP, and MBN (*p* < 0.05) (Table 1). Their interaction significantly affected STN and SOC (*p* < 0.01), as well as STP (*p* < 0.05). In the M0P0 treatment, the contents of SOC, MBC, MBN, MBP were higher than their counterparts in other treatments, partially reaching the significant level (*p* < 0.05). In the M2P0 treatment, plant diversity indices (*H* and *E*) were significantly higher than those in other treatments (*p* < 0.05). In the M1P2 treatment, the contents of STN and STP were significantly higher than those in other treatments (*p* < 0.05) (Table S2).

**FIGURE 4:**
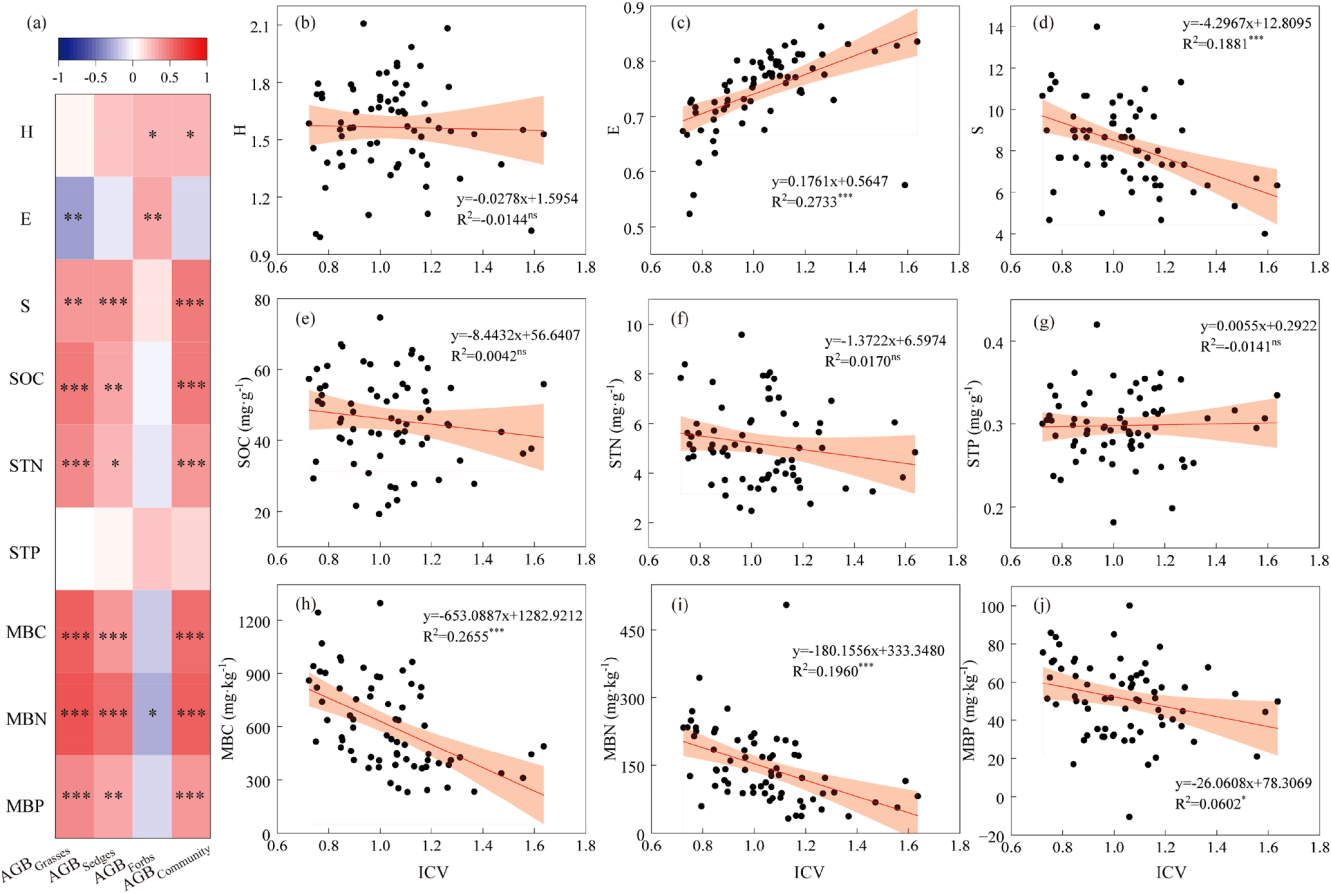
Correlation between plant–soil properties and aboveground biomass (a) and community stability (b–j).

Furthermore, AGB_community_, AGB_grasses_, and AGB_sedges_ were significantly and positively correlated with S, SOC, STN, STP, MBC, MBN, and MBP. Conversely, AGB_grasses_ was significantly and negatively correlated with E. AGB_forbs_ was significantly and negatively correlated with MBN, and significantly positively correlated with H and E detailed correlation coefficients and specific *p*‐values for each relationship are presented in Figure 4a. The results of linear regression analysis showed that ICV was significantly and negatively correlated with S, MBC, and MBN (*p* < 0.001), significantly and negatively correlated with MBP (*p* < 0.05), and significantly and positively correlated with E (*p* < 0.001) (Figure 4b).

### 3.4 Influencing Factors of Plant Community Stability

**FIGURE 5:**
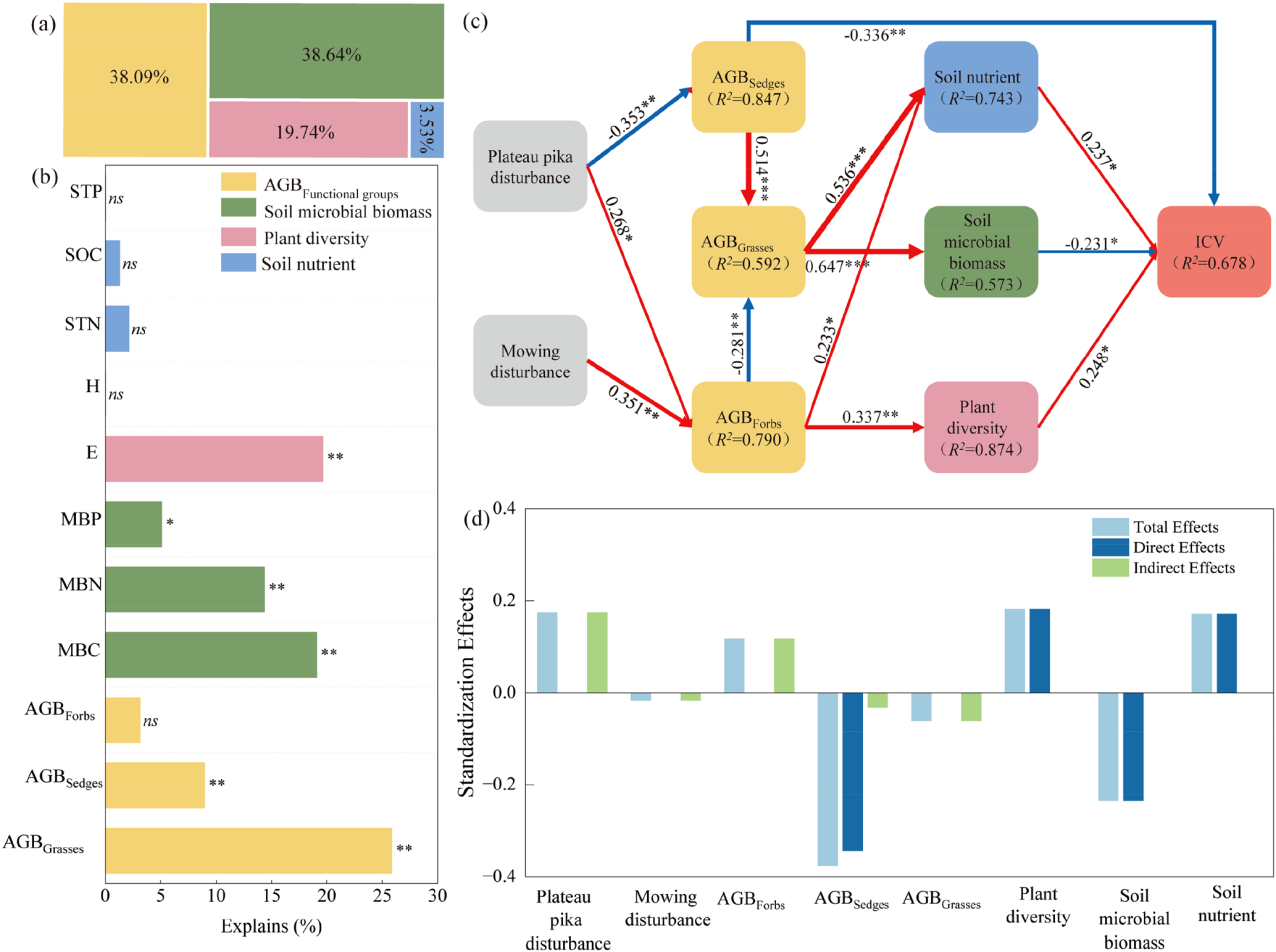
Explanations (a, b) of environmental factors on ICV and paths of affecting plant community stability (c, d). (1) Level of significance: *p < 0.05; **p < 0.01; ***p < 0.001; ns, *p >* 0.05. (2) The model fitting results: *χ* ^2^/df = 1.120, *p* = 0.319; GFI = 0.932; CFI = 0.985; RMSEA = 0.042; RMR = 0.069.

To quantify the relative importance of environmental drivers, variance partitioning analysis (VPA) based on RDA was performed. The results revealed the proportion of total explained variation in ICV attributable to different variable groups, namely, AGB of plant functional groups (38.09%), soil microbial biomass (38.64%); plant diversity (19.74%), and soil nutrients (3.53%) (Figure 5a). These values represent the individual contribution of each group to the total explained variance of ICV. Therefore, AGB and soil microbial biomass were the two paramount variables critical to ICV, whereas soil moisture had a negligible impact on it. The environmental factors with a significant effect on ICV, ranked in descending order of importance, were AGB_grasses_ > *E* > MBC > MBN > AGB_sedges_ (*p* < 0.01). MBP had a significant effect on ICV (*p* < 0.05) (Figure 5b).

The constructed SEM showed that mowing and plateau pika accounted for 67.8% of the variation in plant community stability (Figure 5c,d). They affected plant community stability via three major pathways: (1) They directly affected AGB_sedges_, explaining 84.7% of its variation, thereby significantly reducing ICV (*p* < 0.01); (2) They directly affected AGB_forbs_, explaining 79.0% of its variation. In turn, AGB_forbs_ exerted a significant positive effect on soil nutrients and plant diversity directly (*p* < 0.05), thereby generating a significant positive effect on ICV (*p* < 0.05); and (3) They indirectly affected AGB_grasses_ by influencing AGB_sedges_ and AGB_forbs_, explaining 59.2% of its variation. AGB_grasses_ exerted a significant positive effect on soil nutrients and soil microbial biomass directly (*p* < 0.001), thereby significantly influencing ICV (*p* < 0.05). The modeled results demonstrated that the biomass of plant functional groups played a critical role in mediating and regulating the effects of mowing disturbance and plateau pika disturbance on plant community stability.

## 4 Discussion

### 4.1 Plant Community Composition and Biomass of Functional Groups

The species composition of a community is a crucial determinant of ecosystem traits such as stability, productivity, and nutrient dynamics (Wang et al. 2021). In grassland ecosystems, the formation of plant communities results from complex interactions between plant species and the external environment (Yang et al. 2016). The interaction between plateau pika activity and mowing, as a highly complex and difficult‐to‐quantify disturbance, significantly influences plant community composition (Ren et al. 2012). The results of this study showed that under the joint disturbances of plateau pika and mowing, plant communities in the degraded alpine meadow were dominated by grasses and forbs, although the exact dominant species differed between them. Grasses were primarily dominated by such species as *Poa crymophila* and *Elymus nutans*, while sedge species were predominantly *Carex alatauensis*. The dominant forb species varied significantly with disturbance intensity, but included primarily *Ligularia virgaurea*, *Ajania tenuifolia*, *Elsholtzia densa* Benth., *Ajuga lupulina*, *Lancea tibetica*, and *Euphrasia pectinata* . At an increasingly higher disturbance intensity, the IV of grasses and sedge species gradually decreased, while the IV of forb species gradually increased. These changes were likely associated with selective foraging by plateau pikas initially reducing the height and coverage of palatable forage species, thereby inhibiting their growth and weakening their competitiveness against other species in the same habitat (Niu et al. 2010). This finding aligns well with those of Jia et al. (2014) on the Qinghai‐Tibet Plateau. This phenomenon conforms to the CSR strategy theory (Grime 1977; Marie et al. 2025), namely, conservative (C‐strategy) plants with storage organs (such as rhizomes) gain competitive advantages in a disturbed environment.

Plant functional groups constitute the fundamental basis for the structural composition and functioning of grassland ecosystems (Zhang et al. 2017). They determine grassland ecosystem stability and productivity and respond to external disturbances (Gu et al. 2015; Liu et al. 2016). Vertically, the alpine meadow plant community is composed of grass species in the upper canopy layer, forb species in the intermediate layer, and sedge species in the lower layer. AGB serves as a crucial indicator for assessing grassland health and the stability of grassland ecosystems (Guo et al. 2024). The results of this study indicated that under the sole disturbance of plateau pika, AGB_grasses_ gradually decreased with increasingly heightened disturbance intensity. AGB_forbs_ reached the maximum value at the moderate level of disturbance, while AGB_sedges_ reached the maximum value at the maximum intensity of disturbance. This differential response to disturbance intensity is attributed to the preferential foraging of plateau pika on grassy plants. This selective herbivory suppresses the accumulation of their AGB. Furthermore, grass species are typically dominant owing to their unique strategies in conserving resources (such as via a dense root system). The burrowing and gnawing activities of plateau pikas potentially damage their root systems and tiller structures, potentially impairing the capacity of grasses plants to acquire resources and likely leading to a decline in biomass (Zhang et al. 2018). The intermediate disturbance hypothesis (Connell 1978) posits that moderate disturbance can suppress dominant species (such as grasses), releasing space and resources for the invasion of other species, thereby facilitating the colonization of opportunistic species (forbs). This aligns well with the results of this study, where plateau pika disturbance creates patchy habitats, enabling forbs (such as *Ligularia virgaurea*, *Ajania tenuifolia*) to occupy niches through rapid growth and seed dispersal, achieving the maximum AGB at the level of moderate disturbance. Conversely, sedge plants possess dense belowground storage organs (rhizomes and tubers) and have a low growth rate, both of which enable rapid regeneration after highly intensive disturbance. They also exhibit traits conferring tolerance to trampling and herbivory (e.g., a high fiber content), enabling them to become dominant under a high disturbance stress through stress tolerance (Kong et al. 2020). Under the sole disturbance of mowing, AGB_grasses_ and AGB_forbs_ exhibit completely opposite trends with a heightened disturbance intensity. At the moderate level of mowing, AGB_grasses_ reached the minimum while AGB_forbs_ peaked. This is likely because moderate mowing suppressed primarily the growth of tall grasses in the community. The lowered plants after mowing increased light penetration and created more photosynthetic space, producing a more favorable microhabitat for the growth of forb species in the intermediate layer. This facilitated robust population growth of these species, consequently altering the compositional structure of the alpine meadow community (Ren et al. 2009), a finding consistent with that of Xu et al. (2019). The interaction between mowing disturbance and plateau pika disturbance significantly influenced the biomass of all plant functional groups, particularly AGB_grasses_ and AGB_sedges_. As the primary disturbers in grasslands, mowing and plateau pika regulate the redistribution and transformation of resources by influencing functional groups' diversity, thereby modifying their structure and characteristics (Han et al. 2015).

### 4.2 Plant Diversity and Soil Properties

Plant community diversity quantifies both the evenness of species distribution and the number of species within a community (Wang et al. 2024). It reflects the competitive effects among species and the degree to which the community responds to disturbances (Liao et al. 2015). The more evenly distributed individuals of various species are within a community, the higher its diversity index. The results of this study demonstrated that plateau pika disturbance exerted a significant impact on plant community diversity indices. In the presence of only plateau pika disturbance, plant diversity reached the maximum at the moderate level of disturbance. This outcome also applied to the observed pattern of AGB_forbs_ changes. In the presence of only mowing disturbance, plant diversity increased. However, under the influence of interactive disturbances of both mowing and plateau pika, plant diversity was consistently lower in the disturbed plots than in the reference (control) plots. Moderate plateau pika disturbance created small‐scale heterogeneous habitats within the plots. It suppressed the competitiveness of dominant species (such as grasses) while simultaneously providing niches for annual forbs or pioneer species, thereby enhancing species diversity. This finding aligns with the intermediate disturbance hypothesis (Connell 1978). Mowing enhances diversity by removing AGB (particularly that of taller and dominant grass species), thereby reducing competition for light and nutrients for other dwarf species. This promotes the growth of functional groups of a low stature or a strong regenerative capacity (such as sedges and forbs) (Wei et al. 2022), facilitating niche occupation, accessibility to more natural resources, and a higher plant diversity index. This outcome is consistent with the findings of Yan et al. (2020), confirming that appropriate mowing is beneficial for the sustainable development of grasslands. Conversely, in the presence of both mowing and plateau pika disturbances, they directly reduced AGB and resulted in insufficient accumulation of photosynthetic products, impairing plants' regenerative capacity. Plants must reallocate resources to regenerate after mowing, but the needed resources' depletion could be further exacerbated by plateau pika herbivory (Shi et al. 2022). Dominant functional groups such as grasses and sedges may struggle to recover under this dual disturbance pressure. Although forbs tended to be tolerant of disturbance, their contribution to overall biomass was relatively low, resulting in a decline in total community biomass. In alpine meadows, the sole disturbance of either plateau pika or mowing enhanced diversity by regulating competition among dominant species and resource allocation. However, joint disturbances lead to a decline in diversity due to excessive biomass depletion and consumption and soil degradation at a level beyond the adaptive thresholds of the functional groups.

Soil nutrient content exhibited significant responses to mowing disturbance, plateau pika disturbance, and their interactive effects. The obtained results indicated that mowing disturbance significantly affected the phosphorus content of soil microbial biomass, while plateau pika disturbance significantly influenced soil total phosphorus and the nitrogen content of soil microbial biomass. Their interactions significantly impacted soil organic carbon, total nitrogen, and total phosphorus content. The primary phosphorus source for microbial biomass originates from litter (Zeng et al. 2021). Following mowing, plant residue decreased as the clipped grass was taken out of the ecosystem, reducing the amount of litter that would normally remain in the meadow. This lowers the efficiency of microbial phosphorus immobilization, and changes the content of microbial biomass phosphorus. Plateau pika excreta are rich in nitrogen, potentially increasing soil nitrogen availability in the short term. However, frequent pika disturbances can disrupt microbial community structure (Duan et al. 2024), causing fluctuations in microbial biomass nitrogen. Additionally, pika burrowing may displace low‐phosphorus subsoil to the surface. Plateau pika disturbance increases soil aeration, and promotes microbial decomposition of organic matter. Mowing reduces vegetation cover, and accentuates the direct impact of light and temperature on the soil. The net effects of these synergistic disturbances significantly reduce plant AGB, alter soil structure, and consequently affect the dynamics of soil organic carbon, nitrogen, and phosphorus (Su et al. 2024). This study also reveals that soil organic carbon and soil microbial biomass carbon, nitrogen, and phosphorus in the disturbed plots are all lower than those in the reference plots (control). Soil organic carbon, total nitrogen, and microbial biomass carbon, nitrogen, and phosphorus content all show significant positive correlations with AGB_grasses_ and AGB_sedges_. Grasses and sedges are the dominant functional groups in the alpine meadows. Their high productivity enables them to continuously input organic carbon and nitrogen to the meadow ecosystem through litter and root exudates, promoting the accumulation of soil carbon and nitrogen elements. The dense root systems of these plants also enhance phosphorus activation and absorption through mycorrhizal symbiosis, indirectly maintaining microbial biomass phosphorus (Guo et al. 2018). Under external disturbances, there exists a plant–soil feedback mechanism between grassland plant communities and soil factors. This means that soil factors can strongly influence plant interspecific relationships, community dynamics, and ecosystem functioning (Bever et al. 2012; Xi et al. 2023), resulting in changes to the plant community. Future strategies of grassland management should specifically aim to avoid the concomitant pressure of high‐intensity mowing and high plateau pika densities. While our results highlight the importance of maintaining moderate habitat heterogeneity, caution should be exercised when extrapolating these findings to livestock grazing contexts. Unlike simulated mowing, actual grazing involves additional complex factors such as trampling and nutrient return through excreta, which may further interact with pika activity to influence the ecological functions of alpine meadows.

### 4.3 Mechanism of Disturbances on Plant Community Stability

As one of the most fundamental functions of ecosystems, plant community stability, measured here as the temporal invariability of species densities (ICV), reflects interspecific plant competition and the community's ability to maintain a consistent population structure under environmental perturbations (Shen et al. 2022). The results obtained in this study demonstrated that plant community stability was significantly affected by mowing and plateau pika disturbances, reaching its lowest value at the maximal intensity of both interactive disturbers. The combined dual disturbances exacerbated the depletion of soil nutrients, diminished plant recovery capacity, and caused a consequent decline in plant community stability (Xu et al. 2025). This study also revealed that plant community stability showed a significant negative correlation with both AGB_grasses_ and AGB_sedges_. This occurs because grasses and sedges, as dominant species in the alpine meadows, may suppress the growth of other species through resource competition (such as light and nutrients) when their biomass is too high, thereby reducing community diversity (Jaroszynska et al. 2024). Communities with a low diversity lack functional redundancy, making them more susceptible to instability during environmental fluctuations. This finding has been confirmed by Zhang, Li, et al. (2021). They reported that the expansion of grasses in the Qinghai‐Tibetan Plateau meadows significantly reduced the proportion of forbs, leading to decreased community resilience.

The SEM results indicate that plateau pika and mowing disturbances affect plant community stability primarily through three pathways: (1) Indirect impact on AGB_sedges_, thereby significantly reducing community stability. The dense root systems of sedge plants are known to consolidate topsoil and potentially reduce erosion. Plateau pika disturbance damages their shallow roots and inhibits the formation of new tillers. Mowing disturbance may disrupt energy translocation within sedge plants, potentially weakening the development of overwintering buds. Consequently, post‐disturbance suppression of sedge growth potentially alters soil structure and exacerbates nutrient loss. Furthermore, mowing reduces the biomass of sedge plants and the seasonal resource use efficiency within the plant community (Wei et al. 2024), even though they initiate photosynthesis earlier in spring than other species and exhibit a strong cold tolerance. Therefore, sedge biomass diminished by mowing disrupts the positive plant–soil feedback, resulting in a weakened plant community stability; (2) Both disturbances directly affect AGB_forbs_, which exert significant positive effects on soil nutrients and plant diversity, thereby exerting significant positive effects on plant community stability. Forbs encompass multiple plants, such as nitrogen‐fixing plants and deep‐rooted plants, and the increase in their biomass and species can promote the coexistence of multiple species (Huang et al. 2024). Forb litter typically has a low lignin content, facilitating its rapid decomposition by microbes and releasing nutrients. Increased litter diversity also accelerates soil nutrient cycling (Wei et al. 2023). Additionally, multiple weed species have overlapping ecological functions (Li et al. 2024). Even if some species have declined, others can still function well, buffering the impact of disturbances and sustaining plant community stability; and (3) Both disturbances indirectly affect AGB_grasses_ via their effects on AGB_sedges_ and AGB_forbs_. AGB_grasses_ exert a highly significant positive and direct effect on soil nutrients and soil microbial biomass, thereby significantly influencing plant community stability. Grassy plants are dominant species in alpine meadows. The dense canopy of sedge plants reduces evaporation and preserves moisture, both being conducive to the germination of grasses seeds. Conversely, forbs have the potential to suppress grasses growth potentially through allelopathy and competition via their tall canopy (Zhang, Liu, et al. 2021). Root exudates and litter of grassy plants are reported to provide readily decomposable carbon sources for microbes, likely promoting abundance in microbial biomass nutrients (Li, Fan, and Shangguan 2020). Furthermore, the high cellulose content of grassy plants stimulates the proliferation of cellulolytic bacteria, accelerating organic matter mineralization (Jiang and Song 2010). Moreover, abundant root exudates enhance nutrient availability. High nutrient availability supports multi‐species coexistence, contributing to enhanced plant community stability. Within grassland ecosystems, plant communities comprising multiple species or diverse functional groups tend to exhibit greater stability in light of external disturbances than communities dominated by a mono‐functional group or species (Batbaatar et al. 2021). Within the response mechanism of plant community stability to dual mowing and plateau pika disturbances, the biomass of plant functional groups plays a crucial mediating role. Therefore, consideration of plant functional groups will enhance our understanding of the mechanism underlying community stability of meadow ecosystems in response to external disturbances.

### 4.4 Limitations and Future Research

It should be acknowledged that using mowing as a proxy for grazing focuses primarily on the impact of biomass removal. Since mowing lacks selective herbivory, physical trampling, and nutrient redistribution (manure) inherent to grazing, the ecological response observed in this study may partially differ from actual grazing systems. Future research should integrate these factors for a more comprehensive understanding.

## 5 Conclusion

Plant community composition, functional group biomass, soil characteristics, and community stability respond to the intensity of plateau pika and mowing disturbances differently. Both types of disturbance markedly influenced the IV of plant functional groups, with the IV of grasses reaching its minimum in the M1P2 treatment (medium‐density plateau pikas and heavy mowing). The AGB of grasses and sedges, as well as soil nutrient content, exhibited more pronounced responses to both disturbances and their interactive effects. The contents of SOC, MBC, MBN, and MBP of the undisturbed reference plots were higher than those in the treated plots. Environmental factors with extremely significant effects on community stability were ranked in descending order as AGB_grasses_, E, MBC, MBN, and AGB_sedges_. Plateau pika and mowing disturbances influence plant community stability primarily through three pathways: (1) Both disturbances directly affect the aboveground biomass of sedges, thereby directly reducing community stability; (2) Both disturbances directly affect the AGB of forbs, which exerts a significant positive effect on soil nutrients and plant diversity, thereby exerting a significant positive effect on plant community stability; (3) Both disturbances indirectly affect the AGB of grass plants by influencing the AGB of sedges and forbs. The AGB of grasses exerts a highly significant positive effect on soil nutrients and soil microbial biomass, thereby significantly influencing plant community stability. In summary, the biomass of plant functional groups plays a key mediating role in regulating the impacts of plateau pika and mowing disturbances on plant community stability. Our results underscore that the stability of alpine meadows is highly sensitive to the combined pressures of biological and mechanical disturbances. To ensure long‐term ecosystem resilience, future grassland management must shift from single‐factor mitigation to integrated strategies that avoid dual high‐intensity disturbances (mowing and pikas) and prioritize the maintenance of moderate habitat heterogeneity.

## Author Contributions

**Yu Chai:** conceptualization (lead), data curation (equal), investigation (lead), methodology (equal), visualization (equal), writing – original draft (lead). **Chengyi Li:** conceptualization (supporting), data curation (supporting), investigation (equal), writing – review and editing (equal). **Xinru Du:** data curation (supporting), investigation (supporting), visualization (supporting). **Xilai Li:** conceptualization (equal), data curation (equal), funding acquisition (lead), project administration (lead), resources (lead), writing – review and editing (lead).

## Funding

This study was financially supported by the National Natural Science Foundation of China (U23A20159); Innovation Platform Construction Project of Qinghai Province (2025‐ZJ‐J09); the Higher Education Discipline Innovation Project (the 111 Project of China) (D18013), and the Ecosystem Succession and Management Branch of the World‐Class Discipline of Ecology at Qinghai University.

## Conflicts of Interest

The authors declare no conflicts of interest.

## Supporting information


**Table S1:** Important values of plant species and their functional groups.
**Table S2:** Plant diversity index, soil nutrients and soil microbial biomass in different treatments. (1) Different lowercase letters indicate that the same index is significantly different at the 0.05 level between different treatments. (2) H, Shannon‐Wiener index of plants; E, Pielou evenness index of plants; S, Number of plant species; SOC, Soil organic carbon; STN, Soil total nitrogen; STP, Soil total phosphorus; MBC, Soil microbial biomass carbon; MBN, Soil microbial biomass nitrogen; MBP, Soil microbial biomass phosphorus, the below same.

## Data Availability

The data supporting this study are provided in the supporting tables (Supporting Information S1). These supporting files have been uploaded to the submission system and are accessible to editors and reviewers for peer review purposes.
