# Supplementary material for: Plant Functional Groups Mediated the Effects of Plateau Pika Disturbance and Mowing on the Community Stability of Alpine Meadow Plants
Source: Ecol Evol. 2026 Jun 30;16(7):e73875. doi: 10.1002/ece3.73875 (PMC13316457; doi:10.1002/ece3.73875)
Supplement: Supplementary file 1 — Table S1: Important values of plant species and their functional groups. Table S2: Plant diversity index, soil nutrients and soil microbial biomass in different treatments. (1) Different lowercase letters indicate that the same index is significantly different at the 0.05 level between different treatments. (2) H, Shannon‐Wiener index of plants; E, Pielou evenness index of plants; S, Number of plant species; SOC, Soil organic carbon; STN, Soil total nitrogen; STP, Soil total phosphorus; MBC, Soil microbial biomass carbon; MBN, Soil microbial biomass nitrogen; MBP, Soil microbial biomass phosphorus, the below same. [file ECE3-16-e73875-s001.docx]

**Plant functional groups mediated the effects of plateau pika disturbance and mowing on the community stability of alpine meadow plants**

Yu Chai^a,b^, Chengyi Li^c^, Xinru Du^c^, Xilai Li^a,b^*.

*^a^* *College of Forestry and Grassland, Qinghai University, Xining, Qinghai Province 810016, China; ^b^State Key Laboratory of Plateau Ecology and Agriculture, Qinghai University, Xining 810016, China; ^c^ College of Agriculture and Animal Husbandry, Qinghai University, Xining 810016, China*

***Corresponding author**

E-mail address: [xilai-li@163.com](mailto:xilai-li@163.com)

**Appendix legend**

**Table S1** Important values of plant species and their functional groups

**Table S2** Plant diversity index, soil nutrients and soil microbial biomass under different treatments.

**Note**:1. Different lowercase letters indicate that the same index is significantly different at 0.05 level between different treatments.

2. H: Shannon-Wiener index of plants; E: Pielou evenness index of plants; S: Number of plant species; SOC: Soil organic carbon; STN: Soil total nitrogen; STP: Soil total phosphorus; MBC: Soil microbial biomass carbon; MBN: Soil microbial biomass nitrogen; MBP: Soil microbial biomass phosphorus, the below is same.

**Table S1**

|  | Species of plants | Functional groups | Important value（%） | | | | | | | | |
| --- | --- | --- | --- | --- | --- | --- | --- | --- | --- | --- | --- |
|  |  |  | M0P0 | M0P1 | M0P2 | M1P0 | M1P1 | M1P2 | M2P0 | M2P1 | M2P2 |
| 1 | *Poa crymophila* | grasses | 82.92 | 63.19 | 22.88 | 68.16 | 46.09 | 47.35 | 91.19 | 66.87 | 43.07 |
| 2 | *Elymus nutans* | grasses | 68.90 | 41.19 | 42.49 | 44.23 | 47.25 | 8.52 | 18.61 | 16.64 | 18.77 |
| 3 | *Koeleria cristata* | grasses | 13.84 | 11.31 | 9.34 | 13.06 | 5.69 | 11.45 | — | — | 2.62 |
| 4 | *Stipa capillata* | grasses | 21.54 | 6.60 | — | — | 9.64 | 5.82 | 10.41 | 8.69 | 12.89 |
| 5 | *Kobresia pygmaea* | sedges | 7.40 | 18.04 | 15.25 | 10.22 | 5.77 | 13.59 | 4.21 | 9.78 | 8.65 |
| 6 | *Carex tristachya* | sedges | — | — | — | 20.40 | — | — | 3.95 | — | 16.37 |
| 7 | *Carex alatauensis* | sedges | 21.00 | 35.99 | 42.41 | 4.09 | 13.09 | 24.16 | 15.85 | 16.21 | 10.27 |
| 8 | *Ligularia virgaurea* | forbs | 13.86 | 6.39 | 8.36 | 2.06 | 10.04 | 4.65 | 0.00 | 4.82 | 2.57 |
| 9 | *Pleurospermum szechenyii* | forbs | 8.59 | 2.70 | 2.87 | 11.82 | 8.20 | 3.32 | 16.42 | 3.44 | 4.08 |
| 10 | *Viola rockiana* | forbs | 1.93 | — | — | — | — | — | — | — | — |
| 11 | *Saussurea pulchra* | forbs | 19.66 | 5.80 | 3.48 | 8.12 | 6.97 | 3.89 | 6.81 | 11.45 | 10.17 |
| 12 | *Saussurea salsa* | forbs | — | 15.81 | 12.79 | 14.89 | 16.77 | 11.46 | 14.84 | 18.34 | 2.32 |
| 13 | *Saussurea nutans* | forbs | — | — | 5.68 | 0.85 | 1.59 | — | 1.45 | 0.98 | 0.58 |
| 14 | *Gentiana macrophylla* | forbs | 5.09 | 5.29 | 8.49 | 4.97 | 5.65 | 5.97 | 4.39 | 5.07 | 8.56 |
| 15 | *Microula sikkimensis* | forbs | 0.87 | 3.69 | — | — | 8.03 | 29.77 | — | — | 10.71 |
| 16 | *Anaphalis lactea* | forbs | 1.20 | 4.49 | 4.72 | 4.33 | 6.26 | — | 1.78 | 4.20 | 1.51 |
| 17 | *Ajania tenuifolia* | forbs | 12.95 | 16.00 | 21.81 | 29.41 | 20.54 | 17.36 | 31.43 | 27.68 | 10.46 |
| 18 | *Lagotis integra* | forbs | 7.62 | — | — | — | — | — | — | — | — |
| 19 | *Veronica ciliata* | forbs | 1.92 | 5.68 | 1.76 | 5.02 | 3.01 | 1.06 | — | 3.64 | 9.24 |
| 20 | *Arenaria serpyllifolia* | forbs | 1.65 | 1.78 | — | — | — | 1.61 | — | 3.23 | 7.35 |
| 21 | *Blysmus sinocompressus* | forbs | 1.96 | — | — | — | — | — | — | — | — |
| 22 | *Elsholtzia densa Benth.* | forbs | 2.74 | 5.12 | 16.68 | 15.19 | 26.94 | 22.23 | 9.77 | 26.05 | 22.93 |
| 23 | *Plantago depressa* | forbs | 7.78 | — | — | — | — | — | — | 2.92 | — |
| 24 | *Lamiophlomis rotata* | forbs | 1.73 | — | — | — | — | — | — | — | — |
| 25 | *Oxytropis ochrocephala* | forbs | — | 6.10 | — | 0.89 | 0.71 | 2.32 | 1.64 | 4.97 | 4.00 |
| 26 | *Astragalus membranaceus* | forbs | — | 6.47 | 4.17 | 1.92 | 10.19 | 4.51 | 6.66 | 3.99 | 7.01 |
| 27 | *Taraxacum mongolicum* | forbs | — | 1.12 | — | 2.44 | — | 1.05 | — | — | 3.09 |
| 28 | *Aster tataricus* | forbs | — | 1.12 | — | 1.44 | — | — | 2.04 | — | 0.83 |
| 29 | *Potentilla discolor* | forbs | — | 0.80 | — | — | 1.23 | 0.60 | 1.94 | 3.56 | 2.33 |
| 30 | *Leontopodium leontopodioides* | forbs | — | 3.12 | — | 1.37 | 1.34 | — | — | 1.90 | 1.15 |
| 31 | *Swertia bimaculata* | forbs | — | 2.03 | — | — | — | — | — | — | — |
| 32 | *Ranunculus tanguticus* | forbs | — | 3.44 | 2.70 | 5.15 | 5.24 | 2.24 | — | — | 7.61 |
| 33 | *Ranunculus tanguticus* | forbs | — | — | — | — | — | — | — | 1.79 | — |
| 34 | *Ranunculus membranaceus* | forbs | — | — | — | — | — | 2.18 | — | — | 5.74 |
| 35 | *Ajuga lupulina* | forbs | — | 15.57 | 23.37 | 7.09 | 4.22 | 2.23 | 7.58 | 4.39 | 1.00 |
| 36 | *Pedicularis kansuensis* | forbs | — | 2.50 | 3.59 | — | 1.75 | — | 3.30 | — | — |
| 37 | *Lancea tibetica* | forbs | — | 1.82 | 33.47 | 4.06 | 9.86 | 3.58 | 9.83 | 2.98 | 4.23 |
| 38 | *Stellaria media* | forbs | — | 6.83 | — | — | — | — | — | 4.61 | 4.37 |
| 39 | *Galium trifidum* | forbs | — | — | 4.92 | — | 3.10 | — | 4.99 | — | 11.66 |
| 40 | *Polygonum macrophyllum* | forbs | — | — | 1.17 | 0.98 | — | — | — | 1.70 | — |
| 41 | *Thalictrum cultratum* | forbs | — | — | 2.78 | 1.96 | 3.74 | 5.35 | — | 3.95 | 3.29 |
| 42 | *Cirsium setosum* | forbs | — | — | 4.81 | — | — | — | — | — | — |
| 43 | *Cirsium helenioides* | forbs | — | — | — | — | — | — | — | 0.87 | — |
| 44 | *Halenia elliptica* | forbs | — | — | — | 1.07 | — | — | — | — | 0.48 |
| 45 | *Potentilla longifolia* | forbs | — | — | — | 0.87 | — | — | — | 1.13 | — |
| 46 | *Gentiana sino-ornata* | forbs | — | — | — | 2.00 | — | — | — | 0.85 | — |
| 47 | *Polygonum viviparum* | forbs | — | — | — | 2.95 | 4.26 | — | — | — | — |
| 48 | *Gentianopsis paludosa* | forbs | — | — | — | 2.36 | — | — | — | — | — |
| 49 | *Euphrasia pectinata* | forbs | — | — | — | 6.62 | 8.24 | 31.02 | 5.13 | 16.60 | 25.35 |
| 50 | *Gueldenstaedtia verna* | forbs | — | — | — | — | 1.30 | — | 0.78 | 2.68 | 3.59 |
| 51 | *Aconitum gymnandrum* | forbs | — | — | — | — | 3.29 | 2.52 | — | 8.11 | 1.09 |
| 52 | *Stellera chamaejasme* | forbs | — | — | — | — | — | 0.98 | 3.77 | 1.43 | — |
| 53 | *Chenopodium glaucum* | forbs | — | — | — | — | — | 17.53 | — | 4.50 | 2.50 |
| 54 | *Delphinium grandiflorum* | forbs | — | — | — | — | — | 3.05 | — | — | — |
| 55 | *Lonicera tibetica* | forbs | — | — | — | — | — | — | 4.58 | — | — |
| 56 | *Tibetia himalaica* | forbs | — | — | — | — | — | — | — | — | 0.66 |
| 57 | *Limonium bicolor* | forbs | — | — | — | — | — | — | — | — | 6.91 |

| Treatment | Plant diversity | | |  | Soil nutrient | | |  | Soil microbial biomass | | |
| --- | --- | --- | --- | --- | --- | --- | --- | --- | --- | --- | --- |
|  | H | E | S |  | SOC (mg/g) | STN (mg/g) | STP (mg/g) |  | MBC (mg/kg) | MBN (mg/kg) | MBP (mg/kg) |
| M0P0 | 1.31±0.09ab | 0.70±0.03ab | 6.67±0.48a |  | 60.44±2.08a | 5.54±0.21bc | 0.26±0.01d |  | 642.77±99.67a | 193.83±65.55a | 60.61±3.11a |
| M0P1 | 1.47±0.08a | 0.65±0.05ab | 7.17±1.45a |  | 43.29±3.92b | 6.00±0.51ab | 0.30±0.02bcd |  | 493.02±90.40a | 109.57±20.45b | 41.85±3.67ab |
| M0P2 | 1.08±0.09b | 0.53±0.05b | 5.48±1.15a |  | 39.22±2.98b | 5.22±0.40bcd | 0.28±0.01bcd |  | 501.32±71.10a | 132.87±25.19ab | 58.03±9.65ab |
| M1P0 | 1.42±0.12a | 0.66±0.05ab | 7.89±1.16a |  | 41.66±4.40b | 3.96±0.27d | 0.29±0.01bcd |  | 579.81±74.52a | 132.16±21.92ab | 37.40±7.81b |
| M1P1 | 1.17±0.09ab | 0.57±0.05ab | 6.17±1.15a |  | 39.06±2.48b | 3.89±0.27d | 0.27±0.01cd |  | 581.13±77.53a | 127.91±20.16ab | 39.74±6.19ab |
| M1P2 | 1.18±0.10ab | 0.57±0.05ab | 6.19±1.13a |  | 50.36±3.16ab | 7.21±0.59a | 0.36±0.04a |  | 603.41±70.71a | 141.34±16.91ab | 49.70±6.14ab |
| M2P0 | 1.43±0.08a | 0.71±0.04a | 7.43±0.72a |  | 44.23±4.40b | 6.31±0.60ab | 0.30±0.01bcd |  | 593.26±84.88a | 153.53±18.89ab | 55.16±6.18ab |
| M2P1 | 1.21±0.09ab | 0.59±0.05ab | 6.11±1.19a |  | 47.56±3.01b | 4.57±0.48cd | 0.33±0.01ab |  | 516.06±71.92a | 109.53±22.25b | 51.83±5.61ab |
| M2P2 | 1.27±0.11ab | 0.59±0.05ab | 6.83±1.30a |  | 40.81±4.04b | 4.03±0.20d | 0.32±0.01abc |  | 635.27±90.47a | 141.14±20.66ab | 53.23±5.51ab |

**Table S2**
